# Supplementary material for: A defined mechanistic correlate of protection against Plasmodium falciparum malaria in non-human primates
Source: Nat Commun. 2019 Apr 26;10:1953. doi: 10.1038/s41467-019-09894-4 (PMC6486575; doi:10.1038/s41467-019-09894-4)
Supplement: Supplementary file 2 — Reporting Summary [file 41467_2019_9894_MOESM2_ESM.pdf]

nature research

Corresponding author(s): Alexander Douglas

Reporting Summary

Nature Research wishes to improve the reproducibility of the work that we publish. This form provides structure for consistency and transparency in reporting. For further information on Nature Research policies, see Authors & Referees and the Editorial Policy Checklist.

Statistical parameters

When statistical analyses are reported, confirm that the following items are present in the relevant location (e.g. figure legend, table legend, main text, or Methods section).

n/a	Confirmed	
☐	☒	The exact sample size (*n*) for each experimental group/condition, given as a discrete number and unit of measurement
☐	☒	An indication of whether measurements were taken from distinct samples or whether the same sample was measured repeatedly
☐	☒	The statistical test(s) used AND whether they are one- or two-sided<br>*Only common tests should be described solely by name; describe more complex techniques in the Methods section.*
☒	☐	A description of all covariates tested
☐	☒	A description of any assumptions or corrections, such as tests of normality and adjustment for multiple comparisons
☐	☒	A full description of the statistics including central tendency (e.g. means) or other basic estimates (e.g. regression coefficient) AND variation (e.g. standard deviation) or associated estimates of uncertainty (e.g. confidence intervals)
☐	☒	For null hypothesis testing, the test statistic (e.g. *F*, *t*, *r*) with confidence intervals, effect sizes, degrees of freedom and *P* value noted<br>*Give P values as exact values whenever suitable.*
☒	☐	For Bayesian analysis, information on the choice of priors and Markov chain Monte Carlo settings
☒	☐	For hierarchical and complex designs, identification of the appropriate level for tests and full reporting of outcomes
☐	☒	Estimates of effect sizes (e.g. Cohen's *d*, Pearson's *r*), indicating how they were calculated
☐	☒	Clearly defined error bars<br>*State explicitly what error bars represent (e.g. SD, SE, CI)*

Our web collection on statistics for biologists may be useful.

Software and code

Policy information about availability of computer code

Data collection	Excel (Microsoft) was used for data collation. No custom code was used.
Data analysis	Prism v7 (Graphpad) was used for analysis and graphs. No custom code was used.

For manuscripts utilizing custom algorithms or software that are central to the research but not yet described in published literature, software must be made available to editors/reviewers upon request. We strongly encourage code deposition in a community repository (e.g. GitHub). See the Nature Research guidelines for submitting code & software for further information.

Data

Policy information about availability of data

All manuscripts must include a data availability statement. This statement should provide the following information, where applicable:

- Accession codes, unique identifiers, or web links for publicly available datasets
- A list of figures that have associated raw data
- A description of any restrictions on data availability

All relevant data is presented within the manuscript in graphical form. Raw numerical data is available from the authors on reasonable request.

Field-specific reporting

Please select the best fit for your research. If you are not sure, read the appropriate sections before making your selection.

☒ Life sciences ☐ Behavioural & social sciences ☐ Ecological, evolutionary & environmental sciences

For a reference copy of the document with all sections, see nature.com/authors/policies/ReportingSummary-flat.pdf

Life sciences study design

All studies must disclose on these points even when the disclosure is negative.

Sample size	The primary analysis for efficacy was pre-specified in the study protocol. A calculation of power to detect a 10-fold reduction in the primary endpoint (cumulative parasitaemia to the day of first treatment) was conducted based upon this analysis, using estimates of inter-animal variation derived from our prior experience. Calculated power was 80% with 5 animals per group. A sample size of six was chosen to provide additional power in case of withdrawal of an animal or a non-parametric outcome distribution requiring use of an alternative (also pre-specified) analysis plan.
Data exclusions	No data were excluded.
Replication	In view of the highly statistically significant result and large effect size, replication of the animal study was not felt to be necessary or ethical. Replication of the immunological analyses is described in the figure legends.
Randomization	Animals were randomly assigned to groups. Randomisation was stratified by weight and gender.
Blinding	Staff adjudicating the primary outcome (i.e. protection against malaria, by performing blood film microscopy) were blinded to the allocation of animals to groups. Staff performing subsequent immunological analyses were not blinded.

Reporting for specific materials, systems and methods

Materials & experimental systems

n/a	Involved in the study	
☐	☒	Unique biological materials
☐	☒	Antibodies
☐	☒	Eukaryotic cell lines
☒	☐	Palaeontology
☐	☒	Animals and other organisms
☒	☐	Human research participants

Methods

n/a	Involved in the study	
☒	☐	ChIP-seq
☒	☐	Flow cytometry
☒	☐	MRI-based neuroimaging

Unique biological materials

Policy information about availability of materials

Obtaining unique materials	Reagents used in the study are available from the authors upon reasonable request.

Antibodies

Antibodies used	Antibodies for passive transfer were produced in-house and quality-controlled as described in the manuscript.<br>Antibodies used for ELISA were rabbit anti-monkey polyclonal antibody in HRP-conjugated and AP-conjugated formats (Sigma A2054 and A1929 respectively), HRP-conjugated sheep anti-human C1q polyclonal antibody (Abcam ab46191).
Validation	In view of the lack of validated reagents for this primate species, all validation was performed in-house, using standard methods for ELISA i.e positive control standard curves (via antigen-spiking) and negative controls (no-antigen).

Eukaryotic cell lines

Policy information about cell lines

Cell line source(s)	HEK293F cells (used for monoclonal antibody expression) were obtained from ThermoFisher. HEK293E cells (used for PfRH5 antigen expression for ELISA) were obtained under MTA from the Canadian National Research Council.
Authentication	No specific authentication was performed other than confirmation of expected cell line function i.e. protein production.
Mycoplasma contamination	Testing was not performed as the cells were obtained directly from a commercial supplier
Commonly misidentified lines (See ICLAC register)	N/A

Animals and other organisms

Policy information about studies involving animals; ARRIVE guidelines recommended for reporting animal research

Laboratory animals	Male and female Aotus nancymaae (Nancy Ma's night monkey) from an outbred captive population, aged 16-23 months on day of challenge
Wild animals	N/A
Field-collected samples	N/A
